# Comparison of neuron-based, kernel-based, tree-based and curve-based machine learning models for predicting daily reference evapotranspiration

**DOI:** 10.1371/journal.pone.0217520

**Published:** 2019-05-31

**Authors:** Lifeng Wu, Junliang Fan

**Affiliations:** 1 School of Hydraulic and Ecological Engineering, Nanchang Institute of Technology, Nanchang, China; 2 College of Water Resources and Architectural Engineering, Northwest A&F University, Yangling, China; Politechnika Krakowska im Tadeusza Kosciuszki, POLAND

## Abstract

Accurately predicting reference evapotranspiration (ET_0_) with limited climatic data is crucial for irrigation scheduling design and agricultural water management. This study evaluated eight machine learning models in four categories, i.e. neuron-based (MLP, GRNN and ANFIS), kernel-based (SVM, KNEA), tree-based (M5Tree, XGBoost) and curve-based (MARS) models, for predicting daily ET_0_ with maximum/maximum temperature and precipitation data during 2001–2015 from 14 stations in various climatic regions of China, i.e., arid desert of northwest China (NWC), semi-arid steppe of Inner Mongolia (IM), Qinghai-Tibetan Plateau (QTP), (semi-)humid cold-temperate northeast China (NEC), semi-humid warm-temperate north China (NC), humid subtropical central China (CC) and humid tropical south China (SC). The results showed machine learning models using only temperature data obtained satisfactory daily ET_0_ estimates (on average R^2^ = 0.829, RMSE = 0.718 mm day^−1^, NRMSE = 0.250 and MAE = 0.508 mm day^−1^). The prediction accuracy was improved by 7.6% across China when information of precipitation was further considered, particularly in (sub)tropical humid regions (by 9.7% in CC and 12.4% in SC). The kernel-based SVM, KNEA and curve-based MARS models generally outperformed the others in terms of prediction accuracy, with the best performance by KNEA in NWC and IM, by SVM in QTP, CC and SC, and very similar performance by them in NEC and NC. SVM (1.9%), MLP (2.0%), MARS (2.6%) and KNEA (6.4%) showed relatively small average increases in RMSE during testing compared with training RMSE. SVM is highly recommended for predicting daily ET_0_ across China in light of best accuracy and stability, while KNEA and MARS are also promising powerful models.

## Introduction

Accurate prediction of reference evapotranspiration (ET_0_) is significant for irrigation schedules design, crop growth modeling and agricultural water management [1–5]. Various mathematical models have been proposed to estimate ET_0_ from meteorological variables, among which the FAO-56 Penman–Monteith (FAO-56 PM) equation is suggested by the Food and Agriculture Organization of the United Nations as a reference model in various regions and climates [6], because it considerers both the thermodynamic and aerodynamic items. However, the FAO-56 PM model needs a variety of climatic parameters as model inputs for calculation, e.g., maximum and minimum ambient temperatures, wind speed, relative humidity and net radiation [7–11], which significantly restricts the application of the FAO-56 PM model in many worldwide regions. Therefore, the simplified empirical models with fewer climatic variables is becoming increasingly popular in the absence of compete data [12–15], such as temperature-based models [16], mass transfer-based models [17] and radiation-based models [18]. However, evapotranspiration is a complex and highly nonlinear phenomenon dependent on several climatic parameters. Therefore, it is difficult to establish empirical models that can consider all those complicated processes. In recent years, much attention has been drawn to use alternative techniques such as machine learning models for ET_0_ prediction as a result of their excellent performance in tackling the nonlinear relationship between the model inputs and output [19–21].

The neuron-based machine learning models, i.e. artificial neural networks (ANNs), are the earliest and most widely used models for ET_0_ prediction. [22] compared the multi-layer perceptron (MLP) model and (semi) empirical models to estimate daily ET_0_ in the Basque Country of Spain with different input combinations, and revealed that the MLP model obtained better ET_0_ estimates than the locally calibrated empirical models. [23] also investigated the potential of three ANNs models, including the MLP model, radial basis function neural networks (RBNN) along with generalized regression neural networks (GRNN) for estimating ET_0_ at two weather stations in USA using ambient temperatures, relative humidity, wind speed and solar radiation. It was concluded that the MLP and RBNN models attained satisfactory ET_0_ estimates. [14] evaluated the potential of four ANNs models, including the generalized feedforward (GFF), linear regression (LR), probabilistic neural network (PNN) and MLP models for short-term ET_0_ prediction using forecasted weather data (minimum/maximum temperatures and net solar radiation). It was also found that the MLP model was generally superior to the other three ANNs models. [24] assessed the ANFIS and ANNs models for predicting daily ET_0_ in two locations in South Korea based on temperature, sunshine hours, wind speed and relative humidity. They found that the proposed machine learning models performed well for ET_0_ estimation. [25] compared the capability of three ANNs models (MLP, RBNN and GRNN) with the adaptive neuro fuzzy inference system (ANFIS) and gene expression programming (GEP) for monthly ET_0_ estimation in two locations in the Mediterranean Region of Turkey with only temperature data. They found that the GRNN and GEP models performed better than the other models at one station, while the performance of the RBNN and ANFIS models was best at the other station.

The kernel-based machine learning modes have become increasingly popular for ET_0_ prediction. [26] compared the capability of the kernel-based support vector machine (SVM) with the multiple linear regression (MLR), multiple nonlinear regression (MNLR), ANFIS and empirical models for predicting ET_0_ in the semi-arid regions of Iran. The results indicated that the SVM and ANFIS models performed better than the other models. [13]Wen et al. (2015) predicted daily ET_0_ using the SVM model with few climatic variables in arid China. It was found that the SVM model outperformed the ANNs and empirical models. [27] evaluated the capabilities of the least-squares support vector machine (LS-SVM), multivariate adaptive regression spline (MARS) and M5 model tree (M5Tree) for the estimation of ET_0_ in Mediterranean Turkey, and found that the kernel-based LS-SVM model was superior to the other two models. [28] assessed the potential of the kernel-based extreme learning machine (ELM) to predict daily ET_0_ in Iraq, and found it exhibited good efficiency and generalization performances. [29] estimated daily ET_0_ with the ELM and GRNN models based on air temperatures along in southwestern China. They found that the ELM model was superior to the GRNN model. The kernel-based SVM, LS-SVM and ELM models are also coupled with pre-treatment or optimization algorithms such as wavelet transform (WT) [30] and genetic algorithm (GA) [31] to improve the prediction accuracy. Recently, an improved version of kernel-based machine learning models, i.e., kernel-based nonlinear extension of Arps decline model (KNEA) has been developed and successfully applied in various fields [32–33]. However, this new powerful model has not yet been tested in evapotranspiration studies.

The tree-based machine learning models have recently started to draw researchers’ attention due to their relative simplicity but powerful capability in time-series prediction [34]. [35]evaluated the performance of the M5Tree model for estimating daily ET_0_ in California of USA, and found the M5Tree model gave satisfactory ET_0_ estimates. [36] evaluated the M5Tree and feedforward ANNs models to predict ET_0_ in the arid regions. It was concluded that ET_0_ values predicted by the M5Tree and ANNs models agreed well with the FAO-56 PM values. [37] compared the M5Tree and ANNs models to predict ET_0_ at two sites in USA. They found that the M5Tree model outperformed the ANNs models for estimation of ET_0_ when the input and output data at the target station were not available. [38] compared the random forest (RF) to the GRNN model for prediction of daily ET_0_ in southwestern China, and concluded that the RF model gave better daily ET_0_ estimates than the GRNN model. [39] further used a hybrid RF model with the wavelet algorithm for daily ET_0_ estimation in Southern Iran. They indicated that the new coupled RF model outperformed the classic RF model. [40] explored the performance of two kernel-based models and four tree-based models for daily ET_0_ estimation with limited meteorological data across China. They found that the extreme gradient boosting (XGBoost) and gradient boosting decision tree (GBDT) exhibited similar accuracy and stability compare with the kernel-based SVM and ELM models.

Other machine learning models, such as the curve-based MARS model, have also been applied to predict ET_0_. [27] modeled monthly ET_0_ in Mediterranean Turkey using the MARS, LSSVM and M5Tree models. It was found that the MARS model outperformed the least-squares support vector regression (LSSVR) and M5Tree models. [41] predicted monthly ET_0_ in Iran using the MARS, SVM, GEP and empirical models. The results showed that the MARS and SVM-RBF models were generally superior to the GEP and SVM-Poly models. [42] has also evaluated the performances of the GEP model and the (semi)empirical models for predicting daily ET_0_ in the hyper-arid regions of Iran, and revealed that the superiority of the GEP model for ET_0_ estimation over the (semi)empirical models.

Although the neuron-based, kernel-based, tree-based and curve-based machine learning models have been widely used to predict ET_0_ around the world, their performances are inconsistent in various ET_0_ studies. Particularly, there is still lack of direct and comprehensive comparison of various categories of machine learning models for prediction of ET_0_ in a specific region or country such as China with a vast territory and diverse climates. Due to the limited availability of complete climatic variables, the applicability of these machine learning models for estimation of ET_0_ with more cheaply and reliably measured meteorological variables (e.g. temperature and precipitation) should be explored. In addition, although the utmost attention is usually paid to prediction accuracy when applying machine learning models, model stability is another major factor to consider because unstable models may produce inaccurate ET_0_ estimates if new data are included [34]. Thus, the objectives of this study are to: (1) determine the effects of temperature and precipitation (a variable representing relative humidity to some extent) on the prediction accuracy of daily ET_0_ in different climatic zones of China, and (2) further compare both the prediction accuracy and model stability of eight machine learning models in four categories (MLP, GRNN, ANFIS, SVM, KNEA, M5Tree, XGBoost and MARS) for predicting daily ET_0_ across China as a case study.

## Materials and methods

### Case study and site description

**Fig 1:**
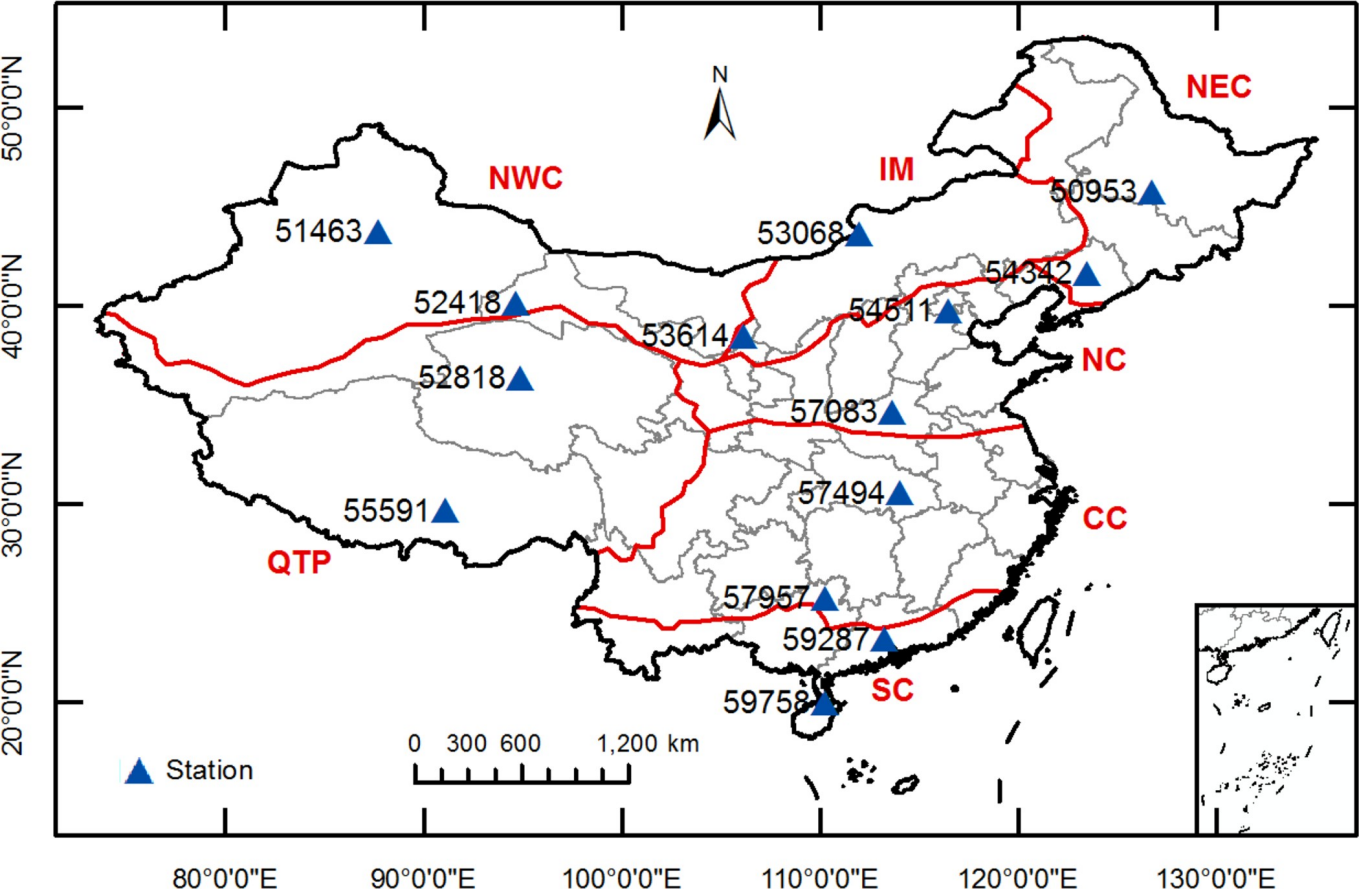
The distribution of different climatic regions across China and the geographical locations of 14 weather stations used in this study. NWC: arid desert of northwest China, IM: semi-arid steppe of Inner Mongolia, QTP: Qinghai-Tibetan Plateau, NEC: (semi-)humid cold-temperate northeast China, NC: semi-humid warm-temperate north China, CC: humid subtropical central China, and SC: humid tropical south China; the South China Sea Islands are presented in the bottom right-hand corner; the same below.

According to multiple-year mean temperatures, precipitation and altitude (Table 1), China is classified as seven climatic regions (Fig 1), i.e. the arid desert of northwest China (NWC), the semi-arid steppe of Inner Mongolia (IM), the Qinghai-Tibetan Plateau (QTP), the (semi-)humid cold-temperate northeast China (NEC), the semi-humid warm-temperate north China (NC), the humid subtropical central China (CC), and the humid tropical south China (SC) [43–44]. The mean daily air temperature in NWC, IM, QTP, NEC, NC, CC and SC varied between -13.2°C and 26.2°C, -10.5°C and 21.1°C, between -9.6°C and 19.8°C, between -19.3°C and 25.7°C, between -4.0°C and 28.4°C, between 1.5°C and 30.7°C and between 3.5°C and 35.6°C, respectively. The average annual precipitation were 269 mm in NWC, 302 mm in IM, 382 mm in QTP, 637 mm in NEC, 658 mm in NC, 1538 mm in CC and 1964 mm in SC.

**Table 1:** The geographical locations and annual mean values (± standard deviation) of meteorological data during 2001–2015 for each of the 14 weather stations used in the present study.

Station ID	Station name	Latitude (N)	Longitude (E)	Altitude (m)	T_max_ (°C)	T_min_ (°C)	P (mm yr^−1^)	ET_0_ (mm d^−1^)
51463	Urumqi	43.8	87.7	935	12.9±13.6	2.8±12.8	271±75.7	2.9±2.3
52418	Dunhuang	40.2	94.7	1139	18.3±14.6	2.2±11.5	42±5.1	3.3±1.7
53614	Yinchuan	38.5	106.2	1111	16.2±10.5	3.6±9.3	203±58.6	2.9±1.8
53068	Erenhot	43.7	112.0	965	11.9±11.4	-2.2±10.7	142±31.2	3.3±1.7
50953	Harbin	45.8	126.8	142	10.2±15.2	-0.9±14.8	479±132.1	2.3±1.8
54342	Shenyang	41.7	123.5	49	14.1±13.4	3.1±12.4	656±150.3	2.4±1.6
54511	Beijing	39.8	116.5	31	18.1±11.4	7.4±10.5	510±145.7	2.9±1.9
57083	Zhengzhou	34.7	113.7	110	20.4±13.5	9.8±11.6	626±154.5	2.9±1.9
52818	Geermu	36.4	94.9	2808	13.1±9.7	-1.2±9.6	44±5.6	3.3±1.8
55591	Lasa	29.7	91.1	3649	16.0±6.0	1.8±7.1	416±132.8	3.4±1.4
57494	Wuhan	30.6	114.1	23	21.4±9.7	13.2±9.2	1241±201.4	2.5±1.7
57957	Guilin	25.3	110.3	164	23.3±8.2	16.0±8.7	1856±248.3	2.7±1.4
59287	Guangzhou	23.2	113.3	41	26.5±6.3	19.0±6.4	1921±270.1	2.7±1.3
59758	Haikou	20.0	110.3	64	28.1±5.7	21.6±6.0	2067±401.7	3.2±1.3

### Data collection and analysis

Long-term daily maximum (T_max_) and minimum (T_min_) temperatures, relative humidity (H_r_), wind speed (U) and horizontal global solar radiation (R_s_) from 2001–2015 were obtained at 14 representative stations across various climatic regions of China (Fig 1). The geographical locations and meteorological values of the 14 stations are presented in Table 1. These meteorological data are provided by the National Meteorological Information Center (NMIC) of China Meteorological Administration (CMA), who has rigorously examined the data quality. The daily data were further excluded if any of the above meteorological data were missing. Overall, missing data accounted for only 0.08% of the database, ranging from 0 to 0.53% at various weather stations.

### FAO-56 Penman–Monteith equation

The FAO-56 Penman–Monteith model was utilized to calculate daily ET_0_ (mm day^-1^) and provide the reference data for the training and testing of machine learning models in this paper:

$$ET_{0}=\frac{0.408\Delta \left(R_{n}-G\right)+\gamma \frac{900}{T_{\mathrm{mean}}+273}U_{2}\left(e_{s}-e_{a}\right)}{\Delta +\gamma \left(1+0.34U_{2}\right)}$$ (1)

where, Rn: net radiation (MJ m^-2^ day^-1^); G: soil heat flux (MJ m^-2^ day^-1^); T_mean_: average ambient temperature (°C); U_2_: wind speed at 2 m height (m s^-1^); e_s_: saturation vapor pressure (kPa); e_a_: actual vapor pressure (kPa); Δ: slope of the vapor pressure curve (kPa °C^-1^); γ: psychrometric constant (kPa °C^-1^). Detailed calculation procedures can be found in [6].

### Machine learning models for predicting daily reference evapotranspiration

#### Multilayer perceptron neural networks (MLP)

The MLP model is one of the widely used ANNs models, which is a feed-forward neural network for nonlinear function approximation. The MLP model consists of three layers: the input, hidden and output layers. The hidden layer often has only one layer, and the number of neural unit has to be determined by the trial and error approach. In the present study, a three-layer neural network was developed: the first layer was input layer, the neural number was equal to the input numbers and the output layer has one neural unit. The MLP model is trained by the Levenberg–Marquardt algorithm, which interpolates between the Gauss–Newton algorithm (GNA) and the gradient descent algorithm. It is more robust than the GNA but still can stick with local, rather than global minima. Further details on the MLP model refer to [4].

#### Generalized regression neural network (GRNN)

The GRNN model is proposed by [45] and is one of the radial basis function neural network (RBF) models. This model can approximate non-linear function of the input and output vectors with a function estimate obtained from the training dataset. It shows a parallel structure and no iterative process is required for model learning between the inputs and outputs. The GRNN model does not need iterative training procedures compared with the back propagation method. Further details on the GRNN model is given by [46].

#### Adaptive neuro-fuzzy inference system (ANFIS)

The ANFIS model is proposed by [47], which is a multi-layer adaptive network coupled with neural networks with the fuzzy inference system. The first-order Sugeno fuzzy model with two fuzzy if-then rules is applied in the ANFIS model to approximate the nonlinear function in this study. The ANFIS model is consisted of five layers: the fuzzification, product, normalization, de-fuzzification and output layers. The model uses different node functions to learn and adjust the parameters in a fuzzy inference system, where the forward and backward passes are applied to decrease the computed errors. More details about the ANFIS model is given by [47].

#### M5 model tree (M5Tree)

The M5Tree model is firstly established by [48], which is a powerful learning method to estimate the true values in a large dataset. It has a series of linear regression functions at the terminal nodes, which develops relationships between the independent variables and a dependent variable. The model firstly constructs a regression tree by splitting the instance space in a recursive manner, and selects the one maximizing the expected errors reduction following all the potential splits. The over-grown trees are then pruned and the sub-trees are then replaced by the linear regression functions. Further details of the M5Tree model refer to [49].

#### Extreme gradient boosting (XGBoost)

The XGBoost model is proposed by [50] and is originated from the idea of "boosting". The XGBoost model integrates all the predictions of a series of “weak” learners to develop a “strong” learner via an additive training process. It is supposed to avoid the over-fitting issue and reduce the computational time. This can be obtained by simplifying the objective functions and combining the predictive and regularization terms, while it maintains optimum computation efficiency at the same time. Parallel calculation is also automatically implemented during the training period. More details about the XGBoost model refer to [50].

#### Support vector machine (SVM)

The SVM model is developed by [51], which is widely used for classification, pattern recognition and regression analysis. The SVM model can estimate the regression on the basis of a set of kernel functions, which are capable of implicitly converting the original, lower-dimensional input dataset to a higher-dimensional feature space. The SVM model has been successfully applied in predicting ET_0_ [31, 41]. The radial basis function (RBF) nonlinear kernel function was utilized in the present study as a result of its outstanding performance for predicting ET_0_ relative to other kernel functions [19](Kisi, 2015), such as linear, polynomial and sigmoid functions. Further information about the SVM model is given by [51].

#### Kernel-based nonlinear extension of Arps decline model (KNEA)

The KNEA model is a newly nonlinear model initially proposed by [32]based on the Arps decline model and kernel method. Compared with the non-parametric and “Black-Box” kernel-based models such as least-squares SVM, the KNEA model is based on the idea of “Grey-Box” and uses the semi-parametric formulation to build the nonlinear models [52]. The kernel-based grey system models are more efficient with small samples [53–54], while the KNEA model performed better with larger samples since samples are not accumulated in the model.

The KNEA model can be described as:

f(x)=af(x−1)+g(u(x))+μ (2)

where *f*(*x*) is the output at this time, *f*(*x*−1) is the output at the last step time. *u*(*x*) represents the factors affecting the output, *g*(*u*(*x*)) can be interpreted as the relationship between *u*(*x*) and *f*(*x*), *μ* is the bias. From this model, we can see that the output of this time is the result of joint action between the output from last time step and the influencing factors at this time.

The nonlinear function *g*(*u*(*x*)) is difficult to determine and can be translated to:

$$g(u(x))=\omega^{T}\varphi (u(x))$$ (3)

This means mapping the original influencing factors into the new space. Therefore, the formula (2) can be written as:

$$f(x)=af(x-1)+\omega^{T}\varphi (u(x))+\mu$$ (4)

Although we still can't solve Eq (4), we can find a very small value so that the difference between the left and right side of the equation is as small as possible:

$$e_{x}=f(x)-af(x-1)-\omega^{T}\varphi (u(x))-\mu$$ (5)

$$\underset{a,\omega ,e}{\min}\varsigma (a,\omega ,e)=\frac{1}{2}a^{2}+\frac{1}{2}{\left\|\omega \right\|}^{2}+\frac{\gamma }{2}\sum_{x=2}^{n}e_{x}^{2}$$ (6)

$$s.t.\;f(x)=af(x-1)+\omega^{T}\varphi (u(x))+\mu +e_{x}$$ (7)

where *γ* is the regularization term, it can control the model smoothness. Like SVM, this optimization problem can be solved by Lagrangian multiplier method:

$$L(a,\omega ,\mu ,e,\lambda )=\varsigma (a,\omega ,e)-\sum_{x=2}^{n}\lambda_{x}\left\{bf(x-1)+\omega^{T}\varphi (u(x))+\mu +e_{x}-f(x)\right\}$$ (8)

where *λ*_*x*_ is the Lagrangian multiplier. The Karush–Kuhn–Tucker (KKT) conditions for optimality of the Lagrangian multiplier method are as follows:

$$\{\begin{array}{ll} \frac{\partial L}{\partial a}=0 & a=\sum_{x=2}^{n}\lambda_{x}f(x-1)\\ \frac{\partial L}{\partial \omega }=0 & \omega =\sum_{x=2}^{n}\lambda_{x}\varphi (u(x))\\ \frac{\partial L}{\partial \mu }=0 & \sum_{x=2}^{n}\lambda_{x}=0\\ \frac{\partial L}{\partial e_{x}}=0 & e_{x}=\lambda_{x}\gamma^{-1}\\ \frac{\partial L}{\partial \lambda_{x}}=0 & f(x)=af(x-1)+\omega^{T}\varphi (u(x))+\mu +e_{x} \end{array}$$ (9)

$$\left(\begin{array}{cc} 0 & 1_{n-1}^{T}\\ 1_{n-1} & \Omega +Q+\frac{1}{\gamma }I_{n-1} \end{array}\right)\left(\begin{array}{c} \mu \\ \lambda \end{array}\right)=\left(\begin{array}{c} 0\\ x_{2|n} \end{array}\right)$$ (10)

where

$I_{n‐1}={\left[1,1,\ldots ,1\right]}_{n-1}^{T}$,

$\lambda_{n-1}={\left[\lambda_{1},\lambda_{2},\ldots ,\lambda_{n-1}\right]}_{n-1}^{T}$,

*x*_2|*n*_ = [*x*(2),*x*(3),⋯,*x*(*n*)]^*T*^,

Ω_*ij*_ = *φ*(*u*(*i*))×*φ*(*u*(*j*)) = *K*(*u*(*i*),*u*(*j*)), where *I*_n-1_ is an *n*−1 dimensional identity matrix with all the diagonal elements as 1 and others as 0. *λ*,*μ* and *a* can be obtained by Eq (9). The Ω_*ij*_ can be employed a kernel function *K*(⋅,⋅), which satisfies the Mercer’s theorem, and a Gauss-type kernel function was selected in the present study. Further information about is given by [32].

$$K(u(i),u(j))=\exp\left[\frac{-{\left(u(i)-u(j)\right)}^{2}}{2\sigma^{2}}\right]$$ (11)

#### Multivariate adaptive regression spline (MARS)

The MARS model is a non-parametric regression approach proposed by [55], which needs no assumption on the relationships between the independent and dependent variables. In the MARS model, a series of coefficients and functions defined as basis functions are used for modeling. The basis function of the MARS model is the outcome of a truncated spline function or multiple spline functions. The number of basis functions and the determination of basis functions are automatically determined by data. Meanwhile, the MARS model integrates the merits of the recursive auto-fractional regression method in dividing spatial regions, projection tracking method in processing high-dimensional data and the advantages of accumulative regression node self-adaptation. Further details of the MARS model refer to [10].

### Input combinations and K-fold cross-validation

**Fig 2:**
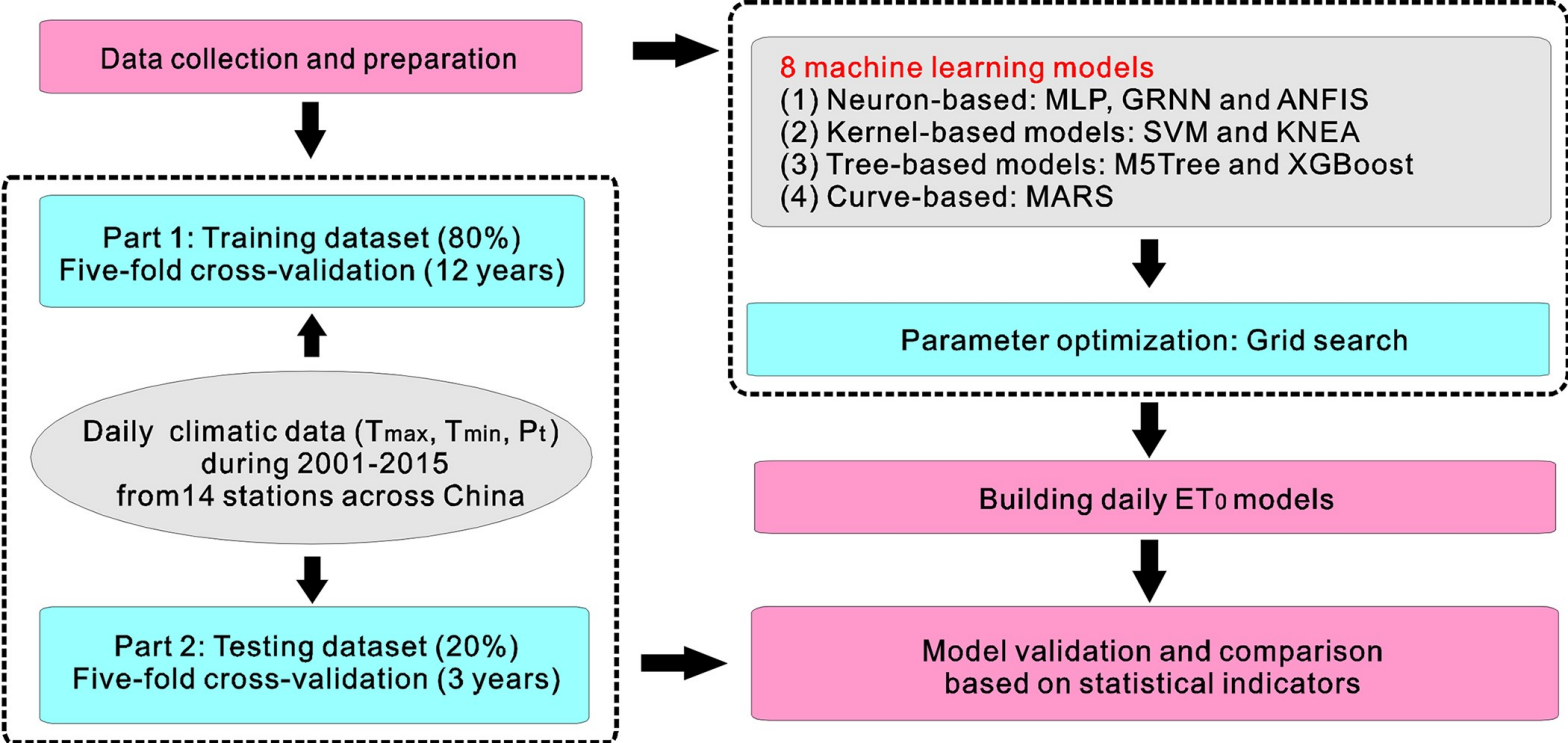
Simple flowchart of the proposed methodology in the present study.

Precipitation is not directly correlated to ET_0_, but it is a manifestation of relative humidity to some extent and may correct the temperature-based ET_0_ models. However, the real amount of precipitation may underestimate or exaggerate its effect on the reduction of daily ET_0_ due to large variation range from 0 mm to even hundreds of mm in humid regions. Therefore, a simple transformed precipitation (P_t_, 1 for precipitation > 0 and 0 for precipitation = 0) was applied here to represent the general effect of precipitation on ET_0_ prediction. Two input combinations of meteorological variables were thus used in the present study to assess the temperature and precipitation effects on daily ET_0_ prediction, i.e., C1: T_max_, T_min_ and R_a_; C2: T_max_, T_min_, and R_a_. The K-fold cross-validation method was applied, where the obtained temperature and precipitation data during 2001–2015 were equally partitioned into five periods. Four periods were used for model training and the last one was used to test the models, which was run over the five various stages (Table 2). The main parameters of the eight machine learning models were optimized by using the grid-search method. Fig 2 presents the simple flowchart of the proposed methodology in the present study.

**Table 2:** The five cross-validation stages involved in the present study.

Cross validation	Training dataset	Testing dataset
S1	2004–2015	2001–2003
S2	2001–2003 and 2007–2015	2004–2006
S3	2001–2006 and 2010–2015	2007–2009
S4	2001–2009 and 2013–2015	2010–2012
S5	2001–2012	2013–2015

### Statistical evaluation

Four common statistical indicators were used in this study to evaluate the models, i.e., RMSE, R^2^, MAE and NRMSE, which can be expressed as [56–57]:

$$R^{2}=\frac{{\left[\sum_{i=1}^{n}\left(Y_{i,m}-{\bar{Y}}_{i,m}\right)(Y_{i,e}-{\bar{Y}}_{i,e})\right]}^{2}}{\sum_{i=1}^{n}{\left(Y_{i,m}-{\bar{Y}}_{i,m}\right)}^{2}\sum_{i=1}^{n}{\left(Y_{i,e}-{\bar{Y}}_{i,e}\right)}^{2}}$$ (12)

$$RMSE=\sqrt{\frac{1}{n}\sum_{i=1}^{n}{\left(Y_{i,m}-Y_{i,e}\right)}^{2}}$$ (13)

$$NRMSE=\sqrt{\frac{1}{n}{\sum_{i=1}^{n}\left(Y_{i,m}-Y_{i,e}\right)}^{2}}/{\bar{Y}}_{i,m}\times 100\%$$ (14)

$$MAE=\frac{1}{n}\sum_{i=1}^{n}\left|Y_{i,m}-Y_{i,e}\right|$$ (15)

where Y_i,m_, Y_i,e_, ${\bar{Y}}_{i,m}$ and ${\bar{Y}}_{i,e}$ are the measured, estimated, mean of measured, mean of estimated reference evapotranspiration, respectively; n is the number of observed data. Higher R^2^ values indicate high prediction accuracy, whereas lower values of RMSE, MAE and NRMSE suggest better model performance. Considering the requirements of the MLP and KNEA models, the raw climatic data were normalized between 0 and 1 as follows:

$$z_{n}=\frac{z_{i}-z_{\min}}{z_{\max}-z_{\min}}$$ (16)

where *z*_*n*_ and *z*_*i*_ is the normalized and raw data; *z*_max_ and *z*_min_ are the minimum and maximum raw data.

## Results and discussion

### Comparison of prediction accuracy of eight machine learning models across China

The statistical results of the eight machine learning models (MLP, GRNN, ANFIS SVM, KNEA, M5Tree, XGBoost and MARS) for estimating daily ET_0_ in NWC (Urumqi and Dunhuang), IM (Yinchuan and Erenhot), NEC (Harbin and Shenyang), NC (Beijing and Zhengzhou), QTP (Geermu and Lasa), CC (Wuhan and Guilin) and SC (Guangzhou and Haikou) under the two input combinations of climatic variables (C1: T_max_, T_min_ and R_a_; C2: T_max_, T_min_, P_t_ and R_a_) during both training and testing periods, are presented in Tables 3–9, respectively.

**Table 3:** Statistical values of the eight machine learning models with different input parameters during training and testing at Urumqi and Dunhuang in the arid desert of northwest China.

Station	Inputs / Model	Training				Testing				(RMSEtes—RMSE_trn_)%	(RMSEC2—RMSE_C1_)_tes_%
		R^2^	RMSE	NRMSE	MAE	R^2^	RMSE	NRMSE	MAE		
			(mm d^−1^)		(mm d^−1^)		(mm d^−1^)		(mm d^−1^)		
Urumqi	**C1 (T**_**max**_**, T**_**min**_**, R**_**a**_**)**										
	MLP1	0.893	0.833	0.283	0.452	0.889	0.841	0.289	0.491	**1.0%**	-
	GRNN1	**0.930**	**0.667**	**0.224**	**0.373**	0.897	0.829	0.279	0.486	24.3%	-
	SVM1	0.893	0.824	0.278	0.458	0.895	0.837	0.281	0.490	1.6%	-
	KNEA1	0.911	0.750	0.252	0.429	**0.898**	**0.822**	**0.277**	**0.473**	9.6%	-
	M5Tree1	0.904	0.779	0.262	0.452	0.889	0.863	0.290	0.511	10.8%	-
	XGBoost1	0.914	0.738	0.248	0.445	0.895	0.836	0.281	0.485	13.3%	-
	ANFIS1	0.907	0.766	0.258	0.443	0.893	0.840	0.282	0.478	9.7%	-
	MARS1	0.897	0.807	0.272	0.476	0.897	0.829	0.278	0.492	2.7%	-
	**C2 (T**_**max**_**, T**_**min**_**, P**_**t**_**, R**_**a**_**)**										
	MLP2	0.915	0.785	0.252	0.432	0.917	0.799	0.256	0.440	**1.8%**	5.0%
	GRNN2	**0.945**	**0.628**	**0.198**	**0.344**	0.905	0.799	0.269	0.465	27.2%	3.6%
	SVM2	0.903	0.782	0.263	0.439	0.903	0.806	0.271	0.457	3.1%	3.7%
	KNEA2	0.926	0.706	0.231	0.395	**0.924**	**0.781**	**0.242**	**0.421**	10.6%	5.0%
	M5Tree2	0.925	0.732	0.232	0.404	0.915	0.812	0.256	0.453	10.9%	5.9%
	XGBoost2	0.940	0.699	0.208	0.364	0.923	0.784	0.244	0.436	12.2%	**6.2%**
	ANFIS2	0.933	0.723	0.220	0.386	0.830	0.789	0.372	0.459	9.1%	6.1%
	MARS2	0.917	0.764	0.244	0.434	0.918	0.784	0.250	0.447	2.6%	5.4%
Dunhuang	**C1 (T**_**max**_**, T**_**min**_**, R**_**a**_**)**										
	MLP1	0.889	0.741	0.224	0.468	0.883	0.750	0.233	0.501	1.2%	-
	GRNN1	**0.923**	**0.603**	**0.181**	**0.392**	0.884	0.766	0.224	0.502	27.0%	-
	SVM1	0.889	0.725	0.218	0.482	0.889	0.732	0.220	0.491	**1.0%**	-
	KNEA1	0.901	0.683	0.205	0.455	**0.890**	**0.729**	**0.219**	**0.490**	6.7%	-
	M5Tree1	0.901	0.681	0.205	0.453	0.881	0.757	0.228	0.516	11.2%	-
	XGBoost1	0.904	0.669	0.201	0.458	0.887	0.737	0.222	0.502	10.2%	-
	ANFIS1	0.897	0.695	0.209	0.465	0.886	0.742	0.223	0.501	6.8%	-
	MARS1	0.888	0.727	0.219	0.487	0.886	0.737	0.222	0.497	1.4%	-
	**C2 (T**_**max**_**, T**_**min**_**, P**_**t**_**, R**_**a**_**)**										
	MLP2	0.893	0.703	0.222	0.487	0.892	0.712	0.223	0.486	1.3%	5.1%
	GRNN2	**0.926**	**0.578**	**0.177**	**0.382**	0.886	0.730	0.223	0.499	26.3%	4.7%
	SVM2	0.899	0.688	0.207	0.460	**0.899**	**0.694**	**0.210**	**0.471**	**0.9%**	5.2%
	KNEA2	0.902	0.649	0.204	0.457	0.897	0.703	0.211	0.477	8.3%	3.6%
	M5Tree2	0.905	0.649	0.201	0.448	0.884	0.726	0.225	0.510	11.9%	4.1%
	XGBoost2	0.923	0.633	0.181	0.418	0.895	0.710	0.214	0.486	12.2%	3.7%
	ANFIS2	0.907	0.662	0.199	0.447	0.885	0.698	0.224	0.498	5.4%	**5.9%**
	MARS2	0.895	0.693	0.212	0.478	0.894	0.705	0.215	0.487	1.7%	4.3%

**Table 4:** Statistical values of the eight machine learning models with different input parameters during training and testing at Yinchuan and Erenhot in the semi-arid steppe of Inner Mongolia.

Station	Inputs / Model	Training				Testing				(RMSEtes—RMSE_trn_)%	(RMSEC2—RMSE_C1_)_tes_%
		R^2^	RMSE	NRMSE	MAE	R^2^	RMSE	NRMSE	MAE		
			(mm d^−1^)		(mm d^−1^)		(mm d^−1^)		(mm d^−1^)		
Yinchuan	**C1 (T**_**max**_**, T**_**min**_**, R**_**a**_**)**										
	MLP1	0.873	0.693	0.249	0.477	0.875	0.696	0.251	0.479	**0.4%**	-
	GRNN1	**0.914**	**0.566**	**0.192**	**0.370**	0.873	0.704	0.238	0.477	24.4%	-
	SVM1	0.881	0.669	0.226	0.444	0.881	0.680	0.230	0.462	1.6%	-
	KNEA1	0.893	0.629	0.213	0.418	**0.882**	**0.680**	**0.230**	**0.456**	8.1%	-
	M5Tree1	0.893	0.632	0.214	0.421	0.863	0.727	0.246	0.494	15.0%	-
	XGBoost1	0.896	0.625	0.212	0.427	0.878	0.688	0.233	0.469	10.1%	-
	ANFIS1	0.888	0.647	0.219	0.436	0.880	0.685	0.232	0.460	5.9%	-
	MARS1	0.879	0.673	0.228	0.458	0.878	0.689	0.233	0.471	2.4%	-
	**C2 (T**_**max**_**, T**_**min**_**, P**_**t**_**, R**_**a**_**)**										
	MLP2	0.892	0.645	0.232	0.449	0.894	0.661	0.238	0.462	2.5%	5.0%
	GRNN2	**0.924**	**0.536**	**0.181**	**0.354**	0.880	0.674	0.231	0.467	25.7%	4.3%
	SVM2	0.885	0.637	0.222	0.439	0.873	0.644	0.238	0.475	**1.1%**	5.3%
	KNEA2	0.903	0.601	0.203	0.413	**0.900**	**0.624**	**0.211**	**0.431**	3.8%	**8.2%**
	M5Tree2	0.906	0.590	0.200	0.406	0.878	0.687	0.232	0.481	16.4%	5.5%
	XGBoost2	0.923	0.567	0.182	0.378	0.898	0.632	0.214	0.440	11.5%	8.1%
	ANFIS2	0.906	0.591	0.200	0.410	0.884	0.651	0.227	0.456	10.2%	5.0%
	MARS2	0.895	0.627	0.212	0.437	0.895	0.642	0.217	0.450	2.4%	6.8%
Erenhot	**C1 (T**_**max**_**, T**_**min**_**, R**_**a**_**)**										
	MLP1	0.896	0.846	0.254	0.555	0.901	0.866	0.259	0.569	2.4%	-
	GRNN1	**0.937**	**0.656**	**0.193**	**0.404**	0.899	0.863	0.253	0.568	31.6%	-
	SVM1	0.900	0.826	0.243	0.538	0.904	0.842	0.247	0.554	**1.9%**	-
	KNEA1	0.916	0.752	0.221	0.486	**0.907**	**0.836**	**0.245**	**0.553**	11.2%	-
	M5Tree1	0.912	0.774	0.228	0.502	0.891	0.896	0.263	0.588	15.8%	-
	XGBoost1	0.916	0.751	0.221	0.499	0.904	0.846	0.248	0.560	12.6%	-
	ANFIS1	0.907	0.788	0.232	0.516	0.901	0.857	0.252	0.558	8.8%	-
	MARS1	0.901	0.820	0.241	0.539	0.905	0.842	0.247	0.556	2.7%	-
	**C2 (T**_**max**_**, T**_**min**_**, P**_**t**_**, R**_**a**_**)**										
	MLP2	0.908	0.795	0.239	0.523	0.914	0.802	0.240	0.532	**0.9%**	7.4%
	GRNN2	**0.943**	**0.624**	**0.184**	**0.385**	0.904	0.826	0.248	0.560	32.4%	4.3%
	SVM2	0.912	0.774	0.228	0.507	0.917	0.791	0.232	0.526	2.2%	6.1%
	KNEA2	0.920	0.716	0.217	0.486	**0.920**	**0.781**	**0.229**	**0.520**	9.1%	6.6%
	M5Tree2	0.919	0.740	0.218	0.485	0.902	0.856	0.251	0.574	15.7%	4.5%
	XGBoost2	0.936	0.686	0.193	0.447	0.917	0.794	0.233	0.532	15.7%	6.1%
	ANFIS2	0.921	0.730	0.215	0.486	0.909	0.792	0.244	0.547	8.5%	**7.6%**
	MARS2	0.910	0.778	0.229	0.517	0.915	0.801	0.235	0.535	3.0%	4.9%

**Table 5:** Statistical values of the eight machine learning models with different input parameters during training and testing at Harbin and Shenyang in the (semi-)humid cold-temperate northeast China.

Station	Inputs / Model	Training				Testing				(RMSEtes—RMSE_trn_)%	(RMSEC2—RMSE_C1_)_tes_%
		R^2^	RMSE	NRMSE	MAE	R^2^	RMSE	NRMSE	MAE		
			(mm d^−1^)		(mm d^−1^)		(mm d^−1^)		(mm d^−1^)		
Harbin	**C1 (T**_**max**_**, T**_**min**_**, R**_**a**_**)**										
	MLP1	0.861	0.675	0.313	0.439	0.868	0.701	0.326	0.457	3.9%	-
	GRNN1	**0.915**	**0.528**	**0.241**	**0.322**	0.873	0.670	0.306	0.434	26.9%	-
	SVM1	0.875	0.643	0.294	0.409	**0.881**	**0.652**	**0.298**	**0.422**	**1.4%**	-
	KNEA1	0.892	0.595	0.272	0.378	0.877	0.663	0.303	0.427	11.4%	-
	M5Tree1	0.891	0.595	0.271	0.377	0.861	0.699	0.319	0.448	17.5%	-
	XGBoost1	0.896	0.586	0.267	0.385	0.879	0.660	0.302	0.431	12.6%	-
	ANFIS1	0.887	0.608	0.278	0.391	0.880	0.658	0.300	0.423	8.2%	-
	MARS1	0.876	0.638	0.291	0.416	0.878	0.661	0.302	0.432	3.6%	-
	**C2 (T**_**max**_**, T**_**min**_**, P**_**t**_**, R**_**a**_**)**										
	MLP2	0.884	0.618	0.286	0.397	0.888	0.639	0.287	0.399	3.4%	**8.8%**
	GRNN2	**0.926**	**0.494**	**0.226**	**0.300**	0.881	0.631	0.297	0.423	27.7%	5.8%
	SVM2	0.893	0.598	0.273	0.376	**0.895**	**0.611**	**0.279**	**0.389**	**2.2%**	6.3%
	KNEA2	0.899	0.556	0.263	0.369	0.894	0.612	0.279	0.396	10.1%	7.7%
	M5Tree2	0.902	0.546	0.258	0.358	0.877	0.654	0.298	0.421	19.8%	6.4%
	XGBoost2	0.923	0.534	0.230	0.335	0.893	0.615	0.281	0.401	15.2%	6.8%
	ANFIS2	0.904	0.560	0.256	0.359	0.888	0.612	0.289	0.404	9.3%	7.0%
	MARS2	0.889	0.601	0.274	0.391	0.890	0.626	0.286	0.407	4.2%	5.3%
Shenyang	**C1 (T**_**max**_**, T**_**min**_**, R**_**a**_**)**										
	MLP1	0.829	0.730	0.320	0.487	0.833	0.743	0.325	0.498	1.8%	-
	GRNN1	**0.896**	**0.571**	**0.238**	**0.367**	0.837	0.724	0.301	0.490	26.8%	-
	SVM1	0.838	0.716	0.298	0.468	0.840	0.725	0.301	0.480	**1.3%**	-
	KNEA1	0.862	0.653	0.272	0.433	**0.849**	**0.699**	**0.291**	**0.474**	7.0%	-
	M5Tree1	0.865	0.648	0.270	0.429	0.827	0.749	0.311	0.504	15.6%	-
	XGBoost1	0.865	0.647	0.270	0.441	0.840	0.719	0.299	0.487	11.1%	-
	ANFIS1	0.857	0.666	0.277	0.448	0.841	0.718	0.299	0.484	7.8%	-
	MARS1	0.840	0.703	0.293	0.478	0.841	0.720	0.299	0.491	2.4%	-
	**C2 (T**_**max**_**, T**_**min**_**, P**_**t**_**, R**_**a**_**)**										
	MLP2	0.851	0.682	0.299	0.459	0.854	0.687	0.301	0.465	**0.7%**	7.5%
	GRNN2	**0.907**	**0.539**	**0.225**	**0.346**	0.846	0.685	0.293	0.478	27.1%	5.4%
	SVM2	0.859	0.666	0.278	0.438	0.861	0.674	0.280	0.449	1.2%	7.0%
	KNEA2	0.871	0.614	0.264	0.424	**0.865**	**0.660**	**0.275**	**0.445**	7.5%	5.6%
	M5Tree2	0.878	0.615	0.256	0.410	0.842	0.703	0.297	0.485	14.3%	6.1%
	XGBoost2	0.898	0.585	0.235	0.391	0.860	0.671	0.279	0.458	14.7%	6.7%
	ANFIS2	0.879	0.614	0.256	0.415	0.860	0.661	0.279	0.454	7.7%	**7.9%**
	MARS2	0.858	0.664	0.277	0.453	0.856	0.685	0.285	0.470	3.2%	4.9%

**Table 6:** Statistical values of the eight machine learning models with different input parameters during training and testing at Beijing and Zhengzhou in the semi-humid warm-temperate north China.

Station	Inputs / Model	Training				Testing				(RMSEtes—RMSE_trn_)%	(RMSEC2—RMSE_C1_)_tes_%
		R^2^	RMSE	NRMSE	MAE	R^2^	RMSE	NRMSE	MAE		
			(mm d^−1^)		(mm d^−1^)		(mm d^−1^)		(mm d^−1^)		
Beijing	**C1 (T**_**max**_**, T**_**min**_**, R**_**a**_**)**										
	MLP1	0.792	0.840	0.311	0.603	0.792	0.841	0.311	0.603	**0.1%**	-
	GRNN1	**0.853**	**0.704**	**0.244**	**0.495**	0.787	0.849	0.294	0.613	20.6%	-
	SVM1	0.795	0.836	0.289	0.580	**0.797**	**0.831**	**0.287**	**0.588**	0.6%	-
	KNEA1	0.817	0.783	0.271	0.558	0.795	0.835	0.289	0.603	6.6%	-
	M5Tree1	0.819	0.779	0.269	0.552	0.774	0.877	0.303	0.633	12.6%	-
	XGBoost1	0.819	0.779	0.270	0.566	0.791	0.842	0.291	0.611	8.1%	-
	ANFIS1	0.809	0.801	0.277	0.574	0.794	0.842	0.292	0.599	5.1%	-
	MARS1	0.797	0.826	0.286	0.596	0.793	0.838	0.290	0.608	1.5%	-
	**C2 (T**_**max**_**, T**_**min**_**, P**_**t**_**, R**_**a**_**)**										
	MLP2	0.818	0.787	0.291	0.576	0.816	0.788	0.292	0.578	**0.1%**	6.3%
	GRNN2	**0.869**	**0.667**	**0.231**	**0.473**	0.799	0.805	0.286	0.601	20.7%	5.2%
	SVM2	0.823	0.774	0.268	0.548	**0.822**	**0.780**	**0.270**	**0.560**	0.8%	6.1%
	KNEA2	0.829	0.758	0.262	0.554	0.821	0.785	0.272	0.573	3.6%	6.0%
	M5Tree2	0.840	0.733	0.254	0.528	0.798	0.829	0.287	0.606	13.1%	5.5%
	XGBoost2	0.860	0.705	0.237	0.508	0.820	0.783	0.271	0.577	11.1%	**7.0%**
	ANFIS2	0.837	0.740	0.256	0.541	0.810	0.784	0.278	0.585	5.9%	6.9%
	MARS2	0.819	0.780	0.270	0.575	0.816	0.791	0.274	0.586	1.4%	5.6%
Zheng- zhou	**C1 (T**_**max**_**, T**_**min**_**, R**_**a**_**)**										
	MLP1	0.785	0.808	0.310	0.594	0.784	0.822	0.316	0.606	1.7%	-
	GRNN1	**0.845**	**0.683**	**0.237**	**0.482**	0.781	0.812	0.282	0.593	18.9%	-
	SVM1	0.791	0.796	0.277	0.561	0.789	0.806	0.280	0.579	**1.3%**	-
	KNEA1	0.811	0.752	0.261	0.541	**0.790**	**0.797**	**0.277**	**0.570**	6.0%	-
	M5Tree1	0.815	0.743	0.258	0.534	0.767	0.840	0.292	0.617	13.1%	-
	XGBoost1	0.814	0.748	0.260	0.549	0.784	0.809	0.281	0.594	8.2%	-
	ANFIS1	0.804	0.766	0.266	0.556	0.787	0.803	0.279	0.583	4.8%	-
	MARS1	0.790	0.793	0.276	0.577	0.788	0.800	0.278	0.584	0.9%	-
	**C2 (T**_**max**_**, T**_**min**_**, P**_**t**_**, R**_**a**_**)**										
	MLP2	0.810	0.757	0.291	0.558	0.808	0.761	0.293	0.561	**0.5%**	7.4%
	GRNN2	**0.862**	**0.645**	**0.224**	**0.458**	0.795	0.767	0.274	0.577	18.9%	5.5%
	SVM2	0.812	0.757	0.263	0.535	0.810	0.762	0.265	0.544	0.7%	5.5%
	KNEA2	0.828	0.718	0.250	0.528	**0.820**	**0.738**	**0.257**	**0.543**	2.8%	7.4%
	M5Tree2	0.836	0.700	0.243	0.509	0.799	0.781	0.272	0.576	11.6%	7.0%
	XGBoost2	0.858	0.681	0.226	0.485	0.818	0.741	0.258	0.549	8.8%	**8.4%**
	ANFIS2	0.835	0.703	0.244	0.518	0.809	0.751	0.265	0.558	6.8%	6.5%
	MARS2	0.815	0.745	0.259	0.552	0.811	0.756	0.263	0.560	1.5%	5.5%

**Table 7:** Statistical values of the eight machine learning models with different input parameters during training and testing at Geermu and Lasa in the Qinghai-Tibetan Plateau.

Station	Inputs / Model	Training				Testing				(RMSEtes—RMSE_trn_)%	(RMSEC2—RMSE_C1_)_tes_%
		R^2^	RMSE	NRMSE	MAE	R^2^	RMSE	NRMSE	MAE		
			(mm d^−1^)		(mm d^−1^)		(mm d^−1^)		(mm d^−1^)		
Geermu	**C1 (T**_**max**_**, T**_**min**_**, R**_**a**_**)**										
	MLP1	0.912	0.544	0.193	0.372	0.916	0.558	0.199	0.386	**2.6%**	-
	GRNN1	**0.935**	**0.466**	**0.147**	**0.305**	0.920	0.545	0.173	0.376	17.0%	-
	SVM1	0.919	0.521	0.165	0.346	**0.923**	**0.535**	**0.170**	**0.363**	2.7%	-
	KNEA1	0.925	0.503	0.159	0.336	0.920	0.541	0.172	0.370	7.6%	-
	M5Tree1	0.927	0.492	0.155	0.328	0.914	0.562	0.178	0.388	14.2%	-
	XGBoost1	0.929	0.485	0.153	0.333	0.921	0.541	0.172	0.373	11.5%	-
	ANFIS1	0.925	0.503	0.159	0.338	0.921	0.537	0.171	0.366	6.8%	-
	MARS1	0.917	0.524	0.166	0.356	0.921	0.542	0.172	0.370	3.4%	-
	**C2 (T**_**max**_**, T**_**min**_**, P**_**t**_**, R**_**a**_**)**										
	MLP2	0.927	0.497	0.176	0.346	0.931	0.520	0.185	0.366	4.6%	6.8%
	GRNN2	**0.951**	**0.436**	**0.128**	**0.288**	0.923	0.512	0.169	0.368	17.4%	6.1%
	SVM2	0.927	0.485	0.157	0.335	**0.937**	**0.494**	**0.154**	**0.344**	**1.9%**	7.7%
	KNEA2	0.934	0.471	0.149	0.323	0.936	0.496	0.157	0.346	5.3%	**8.3%**
	M5Tree2	0.937	0.46	0.145	0.315	0.922	0.528	0.171	0.377	14.8%	6.0%
	XGBoost2	0.941	0.445	0.141	0.294	0.930	0.503	0.163	0.353	13.0%	7.0%
	ANFIS2	0.939	0.471	0.142	0.312	0.886	0.509	0.202	0.368	8.1%	5.2%
	MARS2	0.929	0.485	0.153	0.337	0.932	0.505	0.160	0.354	4.1%	6.8%
Lasa	**C1 (T**_**max**_**, T**_**min**_**, R**_**a**_**)**										
	MLP1	0.885	0.468	0.17	0.347	0.884	0.468	0.170	0.347	**0.0%**	-
	GRNN1	**0.905**	**0.422**	**0.122**	**0.308**	0.885	0.467	0.135	0.345	10.7%	-
	SVM1	0.891	0.452	0.131	0.331	**0.888**	**0.460**	**0.133**	**0.339**	1.8%	-
	KNEA1	0.897	0.441	0.127	0.323	0.885	0.465	0.134	0.344	5.4%	-
	M5Tree1	0.902	0.427	0.123	0.311	0.873	0.491	0.142	0.364	15.0%	-
	XGBoost1	0.904	0.424	0.122	0.315	0.887	0.462	0.133	0.342	9.0%	-
	ANFIS1	0.898	0.437	0.126	0.322	0.883	0.472	0.136	0.344	8.0%	-
	MARS1	0.889	0.455	0.132	0.336	0.886	0.463	0.134	0.344	1.8%	-
	**C2 (T**_**max**_**, T**_**min**_**, P**_**t**_**, R**_**a**_**)**										
	MLP2	0.904	0.424	0.157	0.325	0.902	0.430	0.156	0.324	1.4%	8.1%
	GRNN2	**0.931**	**0.378**	**0.103**	**0.27**	0.895	0.437	0.129	0.332	15.6%	6.4%
	SVM2	0.896	0.411	0.127	0.324	**0.909**	**0.416**	**0.120**	**0.311**	**1.2%**	**9.6%**
	KNEA2	0.911	0.408	0.118	0.304	0.907	0.421	0.122	0.314	3.2%	9.5%
	M5Tree2	0.916	0.396	0.114	0.293	0.894	0.448	0.129	0.336	13.1%	8.8%
	XGBoost2	0.916	0.398	0.115	0.292	0.887	0.433	0.134	0.337	8.8%	6.3%
	ANFIS2	0.918	0.394	0.114	0.294	0.892	0.443	0.131	0.325	12.4%	6.1%
	MARS2	0.905	0.422	0.122	0.317	0.902	0.429	0.124	0.322	1.7%	7.3%

**Table 8:** Statistical values of the eight machine learning models with different input parameters during training and testing at Wuhan and Guilin in the humid subtropical central China.

Station	Inputs / Model	Training				Testing				(RMSEtes—RMSE_trn_)%	(RMSEC2—RMSE_C1_)_tes_%
		R^2^	RMSE	NRMSE	MAE	R^2^	RMSE	NRMSE	MAE		
			(mm d^−1^)		(mm d^−1^)		(mm d^−1^)		(mm d^−1^)		
Wuhan	**C1 (T**_**max**_**, T**_**min**_**, R**_**a**_**)**										
	MLP1	0.858	0.629	0.286	0.432	0.854	0.645	0.294	0.447	2.5%	-
	GRNN1	**0.886**	**0.557**	**0.224**	**0.375**	0.853	0.636	0.256	0.443	14.2%	-
	SVM1	0.864	0.609	0.245	0.410	**0.859**	**0.623**	**0.251**	**0.425**	2.3%	-
	KNEA1	0.871	0.591	0.238	0.401	0.854	0.634	0.255	0.437	7.3%	-
	M5Tree1	0.879	0.573	0.231	0.390	0.844	0.657	0.265	0.457	14.7%	-
	XGBoost1	0.878	0.577	0.232	0.408	0.854	0.636	0.256	0.450	10.2%	-
	ANFIS1	0.870	0.593	0.239	0.405	0.856	0.632	0.254	0.432	6.6%	-
	MARS1	0.862	0.613	0.246	0.422	0.855	0.632	0.254	0.437	3.1%	-
	**C2 (T**_**max**_**, T**_**min**_**, P**_**t**_**, R**_**a**_**)**										
	MLP2	0.883	0.569	0.259	0.397	0.879	0.585	0.262	0.402	2.8%	9.3%
	GRNN2	**0.916**	**0.500**	**0.193**	**0.344**	0.865	0.580	0.245	0.424	16.0%	8.8%
	SVM2	0.890	0.547	0.220	0.377	**0.886**	**0.562**	**0.226**	**0.391**	**2.7%**	9.8%
	KNEA2	0.891	0.543	0.218	0.378	0.883	0.567	0.228	0.396	4.4%	**10.6%**
	M5Tree2	0.901	0.519	0.209	0.363	0.863	0.594	0.247	0.435	14.5%	9.6%
	XGBoost2	0.901	0.510	0.209	0.349	0.882	0.580	0.229	0.402	13.7%	8.8%
	ANFIS2	0.900	0.521	0.210	0.365	0.875	0.567	0.236	0.406	8.8%	10.3%
	MARS2	0.886	0.557	0.224	0.393	0.880	0.574	0.231	0.405	3.1%	9.2%
Guilin	**C1 (T**_**max**_**, T**_**min**_**, R**_**a**_**)**										
	MLP1	0.777	0.725	0.301	0.551	0.776	0.733	0.305	0.558	**1.1%**	-
	GRNN1	**0.826**	**0.634**	**0.230**	**0.480**	0.778	0.718	0.261	0.548	13.2%	-
	SVM1	0.791	0.695	0.252	0.524	**0.787**	**0.705**	**0.256**	**0.533**	1.4%	-
	KNEA1	0.796	0.684	0.248	0.516	0.775	0.724	0.263	0.548	5.8%	-
	M5Tree1	0.812	0.657	0.238	0.496	0.750	0.763	0.277	0.580	16.1%	-
	XGBoost1	0.803	0.673	0.244	0.519	0.774	0.724	0.263	0.559	7.6%	-
	ANFIS1	0.801	0.676	0.245	0.510	0.783	0.711	0.258	0.538	5.2%	-
	MARS1	0.781	0.710	0.258	0.546	0.775	0.723	0.262	0.555	1.8%	-
	**C2 (T**_**max**_**, T**_**min**_**, P**_**t**_**, R**_**a**_**)**										
	MLP2	0.813	0.665	0.276	0.509	0.812	0.670	0.278	0.512	**0.8%**	8.6%
	GRNN2	**0.86**	**0.588**	**0.206**	**0.435**	0.794	0.651	0.251	0.526	10.7%	9.3%
	SVM2	0.83	0.626	0.227	0.47	**0.827**	**0.635**	**0.231**	**0.479**	1.4%	9.9%
	KNEA2	0.821	0.641	0.233	0.489	0.814	0.657	0.239	0.502	2.5%	9.3%
	M5Tree2	0.841	0.606	0.22	0.456	0.790	0.680	0.254	0.531	12.2%	10.5%
	XGBoost2	0.847	0.595	0.216	0.45	0.821	0.645	0.234	0.493	8.4%	**10.9%**
	ANFIS2	0.837	0.612	0.222	0.463	0.808	0.639	0.243	0.504	4.4%	10.1%
	MARS2	0.816	0.651	0.236	0.502	0.813	0.660	0.239	0.508	1.4%	8.7%

**Table 9:** Statistical values of the eight machine learning models with different input parameters during training and testing at Guangzhou and Haikou in the humid tropical south China.

Station	Inputs / Model	Training				Testing				(RMSEtes—RMSE_trn_)%	(RMSEC2—RMSE_C1_)_tes_%
		R^2^	RMSE	NRMSE	MAE	R^2^	RMSE	NRMSE	MAE		
			(mm d^−1^)		(mm d^−1^)		(mm d^−1^)		(mm d^−1^)		
Guangzhou	**C1 (T**_**max**_**, T**_**min**_**, R**_**a**_**)**										
	MLP1	0.737	0.650	0.268	0.498	0.741	0.663	0.273	0.509	2.0%	-
	GRNN1	**0.784**	**0.591**	**0.212**	**0.444**	0.745	0.653	0.234	0.501	10.5%	-
	SVM1	0.755	0.627	0.225	0.469	**0.755**	**0.637**	**0.228**	**0.480**	**1.6%**	-
	KNEA1	0.767	0.611	0.219	0.462	0.753	0.639	0.229	0.488	4.6%	-
	M5Tree1	0.780	0.593	0.212	0.448	0.719	0.681	0.244	0.519	14.8%	-
	XGBoost1	0.775	0.600	0.215	0.459	0.748	0.646	0.232	0.497	7.7%	-
	ANFIS1	0.768	0.610	0.218	0.462	0.747	0.649	0.232	0.491	6.4%	-
	MARS1	0.749	0.633	0.227	0.485	0.750	0.645	0.231	0.495	1.9%	-
	**C2 (T**_**max**_**, T**_**min**_**, P**_**t**_**, R**_**a**_**)**										
	MLP2	0.782	0.590	0.243	0.455	0.786	0.592	0.245	0.457	**0.3%**	10.7%
	GRNN2	**0.837**	**0.520**	**0.183**	**0.391**	0.771	0.592	0.222	0.477	13.8%	9.3%
	SVM2	0.802	0.564	0.202	0.426	**0.801**	**0.567**	**0.207**	**0.440**	0.5%	11.0%
	KNEA2	0.796	0.554	0.204	0.437	0.793	0.570	0.210	0.451	2.9%	10.8%
	M5Tree2	0.818	0.539	0.193	0.408	0.766	0.615	0.224	0.480	14.1%	9.7%
	XGBoost2	0.811	0.534	0.198	0.419	0.797	0.580	0.208	0.447	8.6%	10.2%
	ANFIS2	0.814	0.545	0.195	0.417	0.779	0.579	0.218	0.464	6.2%	10.8%
	MARS2	0.790	0.568	0.208	0.448	0.789	0.572	0.212	0.460	0.7%	**11.3%**
Haikou	**C1 (T**_**max**_**, T**_**min**_**, R**_**a**_**)**										
	MLP1	0.738	0.786	0.262	0.621	0.751	0.798	0.264	0.629	**1.5%**	-
	GRNN1	**0.782**	**0.710**	**0.213**	**0.551**	0.752	0.809	0.242	0.641	13.9%	-
	SVM1	0.750	0.760	0.228	0.595	**0.760**	**0.789**	**0.239**	**0.625**	3.8%	-
	KNEA1	0.742	0.772	0.231	0.602	0.735	0.825	0.247	0.648	6.9%	-
	M5Tree1	0.768	0.735	0.220	0.579	0.729	0.839	0.251	0.660	14.1%	-
	XGBoost1	0.773	0.724	0.217	0.573	0.756	0.802	0.240	0.636	10.8%	-
	ANFIS1	0.764	0.738	0.221	0.580	0.739	0.826	0.247	0.642	11.9%	-
	MARS1	0.741	0.773	0.231	0.611	0.751	0.812	0.243	0.643	5.0%	-
	**C2 (T**_**max**_**, T**_**min**_**, P**_**t**_**, R**_**a**_**)**										
	MLP2	0.805	0.682	0.224	0.524	0.821	0.705	0.236	0.552	**3.4%**	11.7%
	GRNN2	**0.859**	**0.592**	**0.171**	**0.444**	0.789	0.706	0.227	0.597	19.3%	12.7%
	SVM2	0.829	0.632	0.189	0.484	**0.837**	**0.675**	**0.202**	**0.524**	6.8%	14.4%
	KNEA2	0.807	0.668	0.200	0.520	0.814	0.709	0.212	0.556	6.1%	14.1%
	M5Tree2	0.835	0.618	0.185	0.474	0.801	0.734	0.220	0.572	18.8%	12.5%
	XGBoost2	0.805	0.655	0.202	0.529	0.833	0.678	0.203	0.529	3.5%	**15.5%**
	ANFIS2	0.829	0.628	0.188	0.486	0.779	0.716	0.232	0.566	14.0%	13.3%
	MARS2	0.806	0.669	0.200	0.522	0.819	0.709	0.212	0.556	6.0%	12.7%

In terms of R^2^, RMSE, NRMSE and MAE averaged over the fourteen weather stations across China, the GRNN model (on average 0.888, 0.578 mm day^−1^, 0.201 and 0.394 mm day^−1^, respectively), XGBoost model (on average 0.882, 0.612 mm day^−1^, 0.207 and 0.424 mm day^−1^, respectively) and M5Tree model (on average 0.873, 0.626 mm day^−1^, 0.217 and 0.432 mm day^−1^, respectively) generally produced better prediction accuracy in predicting daily ET_0_ than the other machine learning models during training in the whole China. The MLP model (on average R^2^ = 0.850, RMSE = 0.685 mm day^−1^, NRMSE = 0.257 and MAE = 0.476 mm day^−1^) produced the worst performance in all the climatic zones, followed by the MARS model (on average R^2^ = 0.855, RMSE = 0.668 mm day^−1^, NRMSE = 0.232 and MAE = 0.469 mm day^−1^) and SVM model (on average R^2^ = 0.857, RMSE = 0.662 mm day^−1^, NRMSE = 0.232 and MAE = 0.456 mm day^−1^). On the contrary, the SVM model (on average R^2^ = 0.860, RMSE = 0.674 mm day^−1^, NRMSE = 0.234 and MAE = 0.470 mm day^−1^), KNEA model (on average R^2^ = 0.857, RMSE = 0.676 mm day^−1^, NRMSE = 0.236 and MAE = 0.474 mm day^−1^) and MARS model (on average R^2^ = 0.855, RMSE = 0.685 mm day^−1^, NRMSE = 0.237 and MAE = 0.483 mm day^−1^) generally performed better than the other models for daily ET_0_ estimation during the testing period in the whole China. The M5Tree model (on average R^2^ = 0.840, RMSE = 0.716 mm day^−1^, NRMSE = 0.249 and MAE = 0.504 mm day^−1^), MLP model (on average R^2^ = 0.851, RMSE = 0.698 mm day^−1^, NRMSE = 0.259 and MAE = 0.487 mm day^−1^) and GRNN model (on average R^2^ = 0.845, RMSE = 0.696 mm day^−1^, NRMSE = 0.245 and MAE = 0.492 mm day^−1^) performed worst among the machine learning models. These results are in good agreement with other machine learning-based ET_0_ studies. For instance, [58] found that the LS-SVM model yielded accurate ET_0_ estimation in the Changwu County, China. [13] indicated that the SVM model outperformed the ANNs model for the estimation of daily ET_0_ in an extreme arid region of China. [27] found that the LS-SVM model outperformed the MARS and M5Tree models, while the MARS model was superior to the LS-SVM and M5Tree models in cross-station applications. [59] suggested that the SVM model gave better daily ET_0_ estimation than the tree-based assemble models (RF, M5Tree, GBDT and XGBoost) under various input combinations across China.

Specifically, the GRNN model outperformed all the other machine learning models in daily ET_0_ modeling in the seven climatic zones of China during the training period. However, during the testing period, the statistical indicators indicated that the KNEA model had the lowest values of average RMSE, NRMSE, MAE but the highest R^2^ values in NWC (0.757 mm day^−1^, 0.241, 0.476 mm day^−1^ and 0.899, respectively) and IM (0.732 mm day^−1^, 0.229, 0.491 mm day^−1^ and 0.902, respectively). The lowest values of average RMSE, NRMSE, MAE but the highest R^2^ values in QTP (0.477 mm day^−1^, 0.145, 0.339 mm day^−1^ and 0.914, respectively), CC (0.757 mm day^−1^, 0.241, 0.476 mm day^−1^ and 0.899, respectively) and SC (0.757 mm day^−1^, 0.241, 0.476 mm day^−1^ and 0.899, respectively) were obtained by the SVM model. However, the SVM and KNEA models exhibited very close predication accuracy in NEC (on average R^2^ = 0.871, RMSE = 0.659 mm day^−1^, NRMSE = 0.287 and MAE = 0.436 mm day^−1^) and NC (on average R^2^ = 0.806, RMSE = 0.788 mm day^−1^, NRMSE = 0.273 and MAE = 0.576 mm day^−1^). Much lower statistical errors were observed in QTP (on average R^2^ = 0.907, RMSE = 0.489 mm day^−1^, NRMSE = 0.154 and MAE = 0.351 mm day^−1^) compared with those obtained in the other climatic zones (on average R^2^ = 0.850, RMSE = 0.685 mm day^−1^, NRMSE = 0.257 and MAE = 0.476 mm day^−1^). The results suggested that these machine learning models gave higher accuracy in the Qinghai-Tibet Plateau. The RMSE values obtained by these best-performing models in each climatic zone were generally smaller than or close to those obtained in the corresponding regions by previous studies when using only T_max_ and T_min_ data, e.g., by the SVM (0.539 mm day^−1^) and ANN (0.561 mm day^−1^) models in Ejina City of China [13], by ELM (0.444–0.498 mm day^−1^) mode, GANN (0.445–0.499 mm day^−1^) and WNN (0.443–0.641 mm day^−1^) models in the humid region of southwest China using T_max_ and T_min_ [2], by SVM (0.530–0.868 mm day^−1^), M5Tree (0.637–0.953 mm day^−1^) and XGBoost (0.532–0.817 mm day^−1^) models in different climatic zones of China [40].

**Fig 3:**
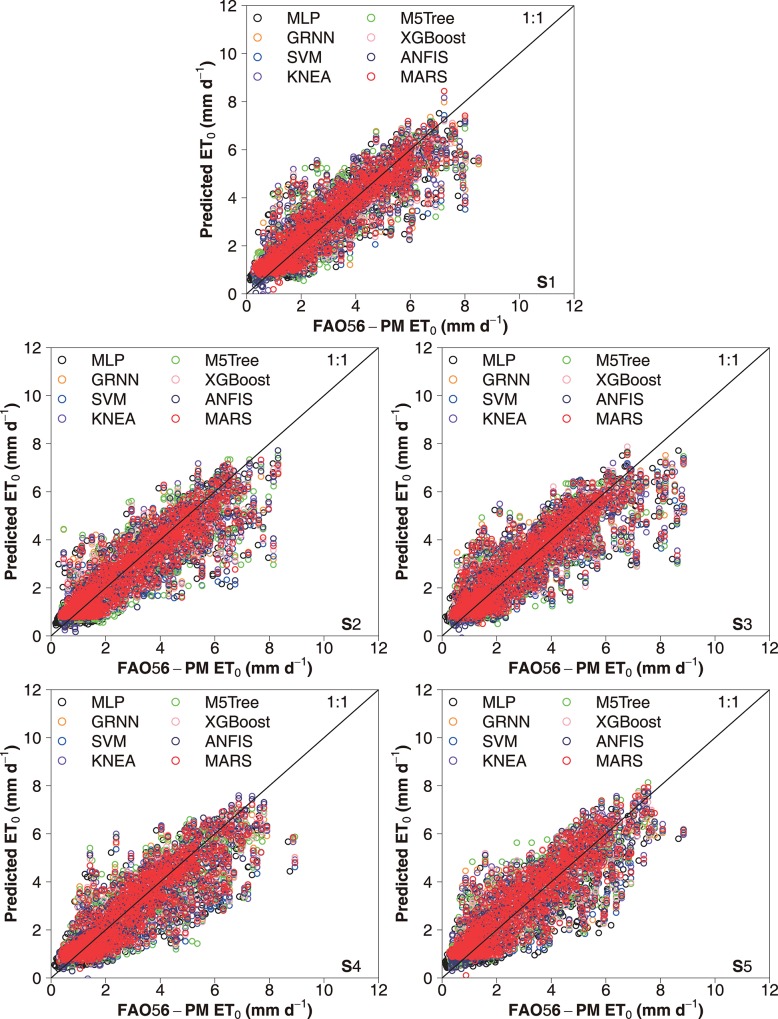
Scatter plots of the ET_0_ values calculated by the FAO-56 PM equation for China’s capital city of Beijing and the values estimated by the eight machine learning models during five cross-validation stages under the input combination of T_max_, T_min_ and R_a_ in the testing stage.

**Fig 4:**
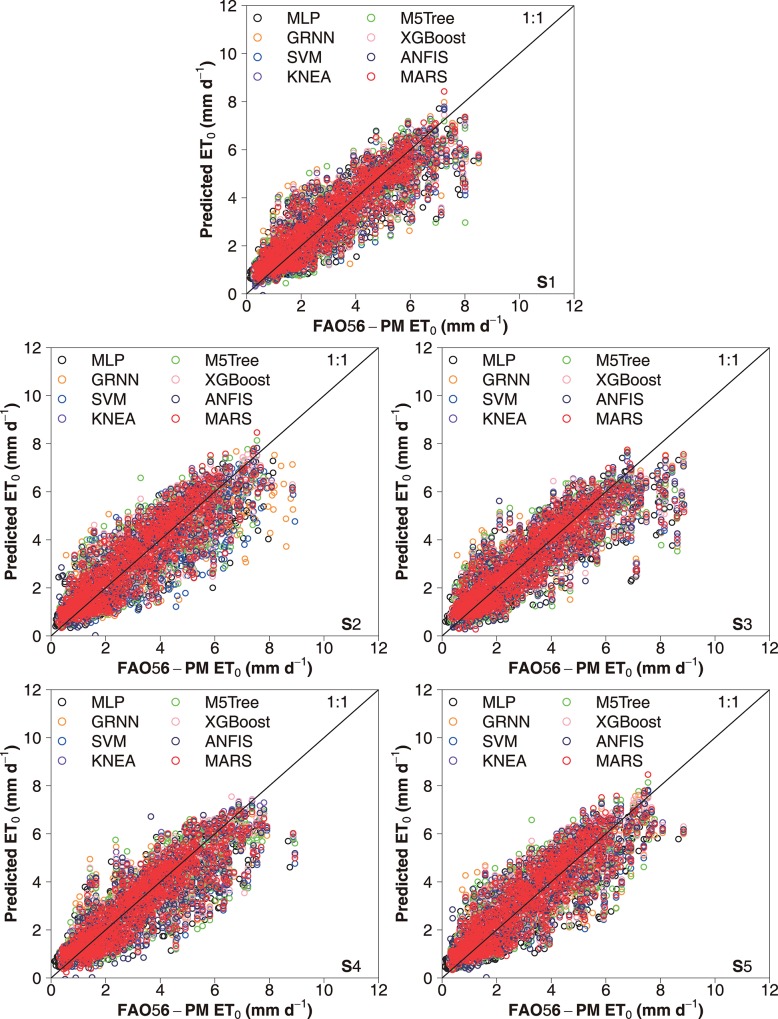
Scatter plots of the ET_0_ values calculated by the FAO-56 PM equation for China’s capital city of Beijing and the values estimated by the eight machine learning models during five cross-validation stages under the input combination of T_max_, T_min_, P_t_ and R_a_ in the testing stage.

**Fig 5:**
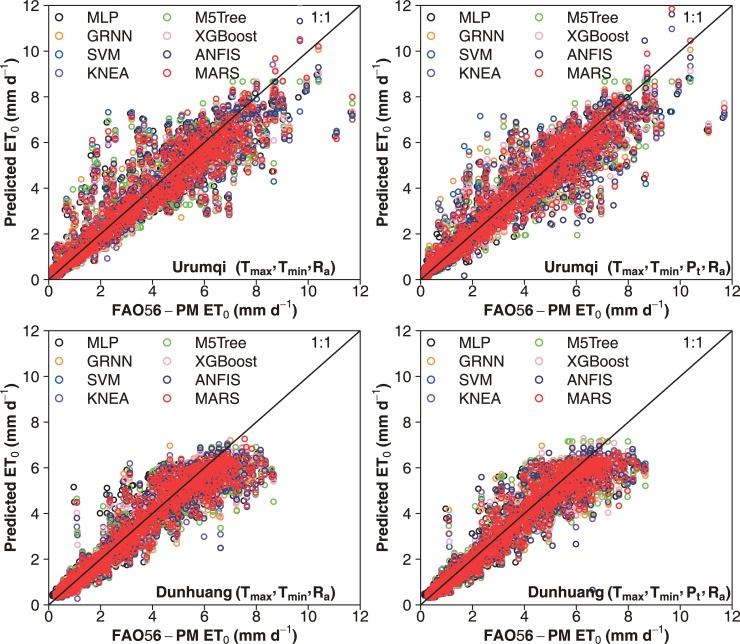
Scatter plots of predicted ET_0_ values using the eight machine learning models against their corresponding FAO56-PM values during testing at Urumqi and Dunhuang in the arid desert of northwest China.

**Fig 6:**
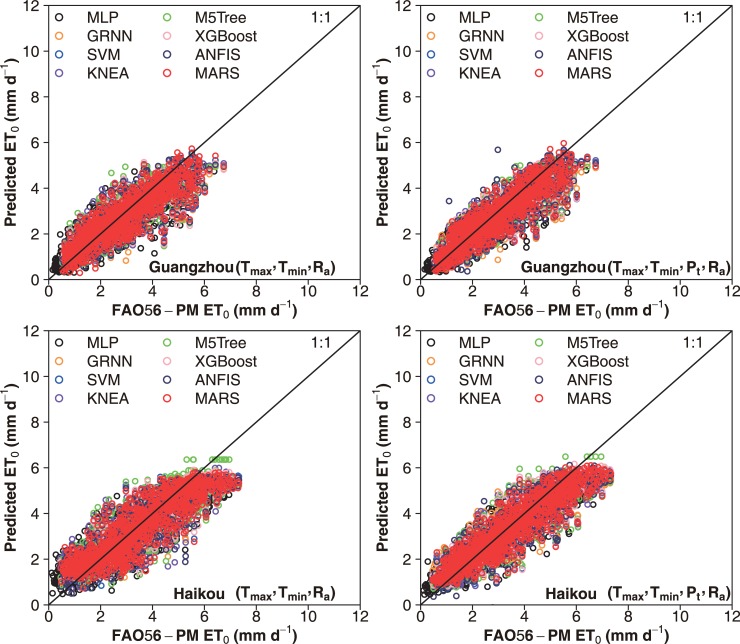
Scatter plots of predicted ET_0_ values using the eight machine learning models against their corresponding FAO56-PM values during testing at Guangzhou and Haikou in the humid tropical south China.

The scatter plots of daily FAO-56 PM ET_0_ values and those predicted by the eight machine learning models for the capital city of China (Beijing) over the five-fold cross validation periods under the two input combinations during testing are presented in Figs 3 and 4, respectively. It can be seen that the selected machine learning models had various prediction accuracies over the five periods. Overall, higher statistical errors were attained during the S4 period (2010–2012), and the S5 period (2013–2015) produced higher prediction accuracy. These differences were largely resulted from the time-series changes in climatic variables among the five periods. This confirms the needs to apply the K-fold cross-validation method for accurately estimating daily ET_0_ in various climates [27, 59]. Nevertheless, the performances of these machine learning models showed the same tendency at various cross-validation stages, with better daily ET_0_ estimates by the SVM, KNEA and MARS models. The dispersion degree of the data points of the SVM, KNEA and MARS models was lower than that of the M5Tree, MLP and GRNN models. Figs 5 and 6 show the scatter plots of estimated values of daily ET_0_ by these machine learning models against the corresponding FAO56-PM values in the arid NWC (Urumqi and Dunhuang) and the humid SC (Guangzhou and Haikou) during the testing period, while the corresponding scatter plots of IM (Yinchuan and Erenhot), NEC (Harbin and Shenyang), NC (Beijing and Zhengzhou), QTP (Geermu and Lasa) and CC (Wuhan and Guilin) can be found in S1–S5 Figs. It is seen that the M5Tree, MLP and GRNN models exhibited more scattered estimates compared with the other models. The daily ET_0_ values predicted by the SVM, KNEA and MARS models were more close to the corresponding FAO-56 PM values and generally showed the same tendency, which further confirmed the superiority of the SVM, KNEA and MARS for daily ET_0_ estimation across China.

### Comparison of prediction accuracy of eight machine learning models under two input combinations

As seen from Tables 3–9, the predicted ET_0_ values differed significantly under the two input combinations of climatic variables (C1: T_max_, T_min_ and R_a_; C2: T_max_, T_min_, P_t_ and R_a_) during training and testing. Generally, machine learning models with the input combination of T_max_, T_min_, P_t_ and R_a_ (on average R^2^ = 0.879, RMSE = 0.613 mm day^−1^, NRMSE = 0.214 and MAE = 0.426 mm day^−1^ during training; R^2^ = 0.862, RMSE = 0.663 mm day^−1^, NRMSE = 0.235 and MAE = 0.469 mm day^−1^ during testing) obtained better prediction accuracy compared with those with the input combination of T_max_, T_min_ and R_a_ (on average R^2^ = 0.844, RMSE = 0.666 mm day^−1^, NRMSE = 0.232 and MAE = 0.467 mm day^−1^ during training; R^2^ = 0.829, RMSE = 0.718 mm day^−1^, NRMSE = 0.250 and MAE = 0.508 mm day^−1^ during testing) in the whole China. These statistical results were generally similar to those obtained by various machine learning models with only T_max_ and T_min_ data in previous studies [2, 13, 40]. However, the incorporation of P_t_ as input parameter to the machine learning models introduced an average decrease in RMSE by 7.7% and 7.6% during the training and testing periods, respectively. Specifically, an average decrease in RMSE by 4.8%, 6.0%, 7.4%, 6.5% and 6.5% were obtained during the testing period by considering P_t_ in the machine learning models in NWC, IM, QTP, NEC and NC, respectively. However, much higher decreases in RMSE were obtained in CC (by 9.7%) and SC (by 12.4%) by machine learning models with the input combination of T_max_, T_min_, P_t_ and R_a_ compared with those with the input combination of T_max_, T_min_ and R_a_. These results indicated that the incorporation of precipitation information in machine learning models can improve the prediction accuracy of daily ET_0_, particularly in the subtropical and tropical humid regions (Tables 3–9). [59]have found that the prediction accuracy of empirical and machine learning models for estimating daily global solar radiation can be much improved by considering precipitation as an input, because it was a manifestation of cloud cover and could correct the temperature-based models by considering its effects on the radiation reduction. This can also explain why the performance of machine learning models for daily ET_0_ prediction from daily minimum/maximum temperature can be enhanced when the information of precipitation was further included.

### Comparison of model stability of eight machine learning models

As illustrated earlier, the GRNN, XGBoost and M5Tree models outperformed the corresponding MLP, SVM, KNEA, ANFIS and MARS models for predicting daily ET_0_ in the whole China during the training period in terms of R^2^, RMSE, NRMSE and MAE (Tables 3–9). However, the SVM, KNEA and MARS models produced better daily ET_0_ estimates compared with the other machine learning models during the testing period. The percentage increase in RMSE during testing relative to RMSE during training by the eight machine learning models for estimating daily ET_0_ in NWC (Urumqi and Dunhuang), IM (Yinchuan and Erenhot), NEC (Harbin and Shenyang), NC (Beijing and Zhengzhou), QTP (Geermu and Lasa), CC (Wuhan and Guilin) and SC (Guangzhou and Haikou) under the two input combinations are also shown in Tables 3–9. The model stability is also an essential factor to consider for predicting more accurate and reliable daily ET_0_. These tables suggested that the SVM, MLP and MARS models were the most stable models with the consistently small percentage increase in RMSE during testing relative to that during training in all the climatic zones of China (on average 1.9%, 2.0% and 2.6%, respectively). The KNEA and ANFIS models also exhibited relatively smaller increase in testing RMSE (on average 6.4% and 7.8%, respectively). However, the GRNN, M5Tree and XGBoost models exhibited the much larger increases in testing RMSE (on average 20.1%, 14.5% and 12.0%, respectively). These increase indicated the instability of the GRNN, M5Tree and XGBoost models as they introduced high decreases in model performances when including new dataset. [40]showed that the kernel-based SVM and ELM models were more stable compared with the tree-based RF, M5Tree and XGBoost models for the estimation of daily ET_0_. [34]also found that the RF and bagging models showed greater increases in RMSE during testing compared with the SVR and gradient models when predicting global solar radiation. These suggest that the kernel-based machine learning models (e.g., SVM, ELM and KNEA) are generally more stable than the tree-based models (RF, M5Tree and XGBoost).

### Comprehensive evaluation of eight machine learning models

The SVM, KNEA and MARS models outperformed the other machine learning models in daily ET_0_ modeling in terms of prediction accuracy during the testing period. Considering the model stability, the SVM, MLP and MARS exhibited very small percentage increase in RMSE during testing (< 3.0%), while the KNEA and ANFIS models showed relatively small increase in testing RMSE (< 8.0%). The SVM model exhibited the best combination of prediction accuracy and model stability among the eight machine learning models, while the KNEA and MARS model also provided satisfactory combination of prediction accuracy and model stability. Comprehensively considering the prediction accuracy and model stability, the SVM, KNEA and MARS models are recommended for estimating daily ET_0_ using only temperature and precipitation data across various climatic regions of China and maybe elsewhere in similar climates.

## Conclusions

The performance of eight machine learning models in four categories, e.g. neuron-based (MLP, GRNN, ANFIS), kernel-based (SVM, KNEA), tree-based (M5Tree, XGBoost) and curve-based (MARS) models, for the estimation of daily ET_0_ were compared based on only temperature and precipitation data during 2001–2015 obtained from 14 representative stations across various climatic zones of China. The results showed that the machine learning models using only temperature attained satisfactory daily ET_0_ estimation. The prediction accuracy was further improved across China when information of precipitation was considered, especially in the (sub)tropical humid regions. This indicates that precipitation is a manifestation of relative humidity to some extent and can correct the temperature-based ET_0_ models. The kernel-based SVM, KNEA and curve-based MARS models generally gave more accurate daily ET_0_ estimates than the other models for, with the best performance by KNEA in NWC and IM, by SVM in QTP, CC and SC, as well as a similar best performance by them in NEC and NC. The SVM, MLP, MARS and KNEA models showed relatively small percentage increase in RMSE during testing over the training one. Comprehensively considering both prediction accuracy and model stability, SVM is highly suggested, while KNEA and MARS are also alternative models for predicting daily ET_0_ in various climatic regions of China. The satisfactory performances of these proposed machine learning models with ambient temperatures and transformed precipitation indicates that it is possible for near-future prediction of daily ET_0_ using public weather forecasts, including daily maximum and minimum temperatures and whether there is precipitation or not. Nevertheless, more study is needed to explore the performances of the proposed machine learning models at varying temporal scales or in various climatic regions.

## Supporting information

S1 Fig Scatter plots of predicted ET_0_ values using the eight machine learning models against their corresponding FAO56-PM values during testing at Yinchuan and Erenhot in the semi-arid steppe of Inner Mongolia. (EPS)

S2 Fig Scatter plots of predicted ET_0_ values using the eight machine learning models against their corresponding FAO56-PM values during testing at Harbin and Shenyang in the (semi-)humid cold-temperate northeast China. (EPS)

S3 Fig Scatter plots of predicted ET_0_ values using the eight machine learning models against their corresponding FAO56-PM values during testing at Beijing and Zhengzhou in the semi-humid warm-temperate north China. (EPS)

S4 Fig Scatter plots of predicted ET_0_ values using the eight machine learning models against their corresponding FAO56-PM values during testing at Geermu and Lasa in the Qinghai-Tibetan Plateau. (EPS)

S5 Fig Scatter plots of predicted ET_0_ values using the eight machine learning models against their corresponding FAO56-PM values during testing at Wuhan and Guilin in the humid subtropical central China. (EPS)

S1 Data (RAR)
